# Aquaporin and Vascular Diseases

**DOI:** 10.2174/157015910791233196

**Published:** 2010-06

**Authors:** Carla Loreto, Ester Reggio

**Affiliations:** 1 Department of Anatomy, Diagnostic Pathology, Forensic Medicine, Hygene and Public Health, University of Catania, Via S. Sofia 87, 95123 Catania, Italy; 2 Department of Neuroscience, University of Catania, Via S. Sofia 87, 95123 Catania, Italy

**Keywords:** Aquaporin, CNS, Vascular diseases.

## Abstract

Aquaporins (AQP) are family of water channels found in several epithelial and endothelial cells, whose recent identification has provided insights into water transport in several tissues, including the central nervous system (CNS). Since brain edema continues to be the main cause of death from several CNS diseases, such as stroke, much of the interest in AQPs and their functional contribution to the water balance is due to their possible role in clearing edema water from the brain and in managing hydrocephalus and benign intracranial hypertension, suggesting that they could be targets for future treatments of various brain conditions, particularly vascular diseases. AQPs also seem to be involved in  cell migration, and a mechanism of AQP-facilitated cell migration has been proposed where local osmotic gradients created at the tip of the lamellipodium drive water influx, facilitating lamellipodial extension and cell migration. AQP-facilitated cell migration was also detected in tumour cells, suggesting that it may have an important role in tumour angiogenesis and spread, and accounting for AQP expression in many tumour cell types and for correlations found between AQP expression and tumour stage in some tumours.

## 1 INTRODUCTION

Aquaporins (AQP) are family of homologous water channels found in several epithelial and endothelial cell types involved in fluid transport. Expression of AQP1, AQP4 and AQP9 is found in the brain and in other tissues in the body.

AQP1 is strongly expressed in the majority of microvascular endothelia outside the brain, as well as in endothelial cells in cornea, intestinal lacteals, renal proximal tubular epithelium, and other tissues [94, 98, 109]. Immunoreactivity for AQP1 has been detected in the capillary endothelium of pig ovary [87] and of rat parotid and submandibular gland [30], in the microvascular endothelia of mouse lungs and airways, and in toad subepidermal capillary endothelium [96].

AQP4 is the predominant AQP in the central nervous system (CNS) [42]. It is also found at other sites such as the basolateral membrane of the collecting duct epithelium, with a relatively greater expression in the inner medullary collecting duct [97], the basolateral membrane of surface epithelial cells in the airways, and salivary gland ductal epithelia [94].

High-level AQP9 expression has been documented in the liver [22, 92], with polarization of the protein to the hepatocyte plasma membrane facing the sinusoids [22, 64, 67].

## 2 AQP1

AQP1 is selectively and constitutively expressed in the CNS, in the apical surface of the choroid plexus (CP) epithelium and, to a lesser extent, in the basolateral membrane and endothelia, where it acts both as a water channel and as a cGMP-gate ion channel [12, 66]. AQP1 also transports nitric oxide across the cell membrane [34]. Its expression in various vascular and non-vascular endothelia suggests the hypothesis of its involvement in osmotically-driven transendothelial water movement [98]. In mice, targeted disruption of the AQP1 gene induced a decrease in cerebrospinal fluid (CSF) production and in intracranial pressure (ICP), indicating a primary role for it in CSF secretion within the CNS [69]. AQP1 levels are low in normal brain capillary endothelial cells [20, 66], and no expression has been detected in primary astrocyte cultures from rodents [20].

Atrial natriuretic peptide (ANP), a CSF modulator, has been implicated in AQP1 channel function regulation. Notably, activation of the AQP1 cation channel by ANP-activated cGMP signaling reduces the net fluid flow from the basolateral to the apical membrane in confluent layers of cultured rat CP. The effect is reversed by AQP1 ion channel blockers [12].

A role for AQP1 as an osmosensor has also been surmised [35], whereby AQP1 would enable the choroid cells to sense CSF osmolarity, resulting in transport rate adjustments. Sporadic AQP1 distribution has been described in the basolateral membrane and at a much lower level in the apical membrane [74].

AQP1 is expressed in brain capillary endothelial cells from brain tumours that are not surrounded by astrocytic end-feet [80], suggesting that the latter may signal adjacent endothelial cells to switch off its expression.

In pathological conditions AQP1 is also expressed elsewhere; its upregulation has been documented in cerebral vessel endothelia, indicating a role for it in clearing edema water from the brain [25]. These data suggest that AQP1 could be a target for future treatments of various brain conditions, particularly vascular diseases [111].

## 3 AQP4

AQP4 is the predominant AQP in the CNS [42], where it participates in brain water homeostasis by acting as a key constituent of the blood-brain barrier (BBB) and the blood-CSF barrier [65]. It is expressed in pericapillary astrocyte foot processes, external glial limiting membrane, ependyma, and subependymal internal glial limiting membrane [65, 76].

Marked changes in its levels have been described during brain development [102]. In particular, AQP4 up-regulation in rat cerebellar glia begins in the second postnatal week [109]. mRNA and protein signals increase slightly in the course of the first week, rising more than 10-fold during the second week (postnatal day [PN] 7-14), and reaching ca. 60% of adult levels by week 4. The change seems to parallel the time course of a reduction in the volume fraction of the extracellular space, which starts on PN6-7 and accelerates between PN10 and PN21. Its pattern of expression suggests that AQP4 may contribute to the development of brain water homeostasis and that it may begin to exert its effects from the second week of postnatal life [102].

Moreover, the presence of AQP4 in the end-foot membrane has been shown to depend on the presence of proteins in the basal lamina such as agrin, α-dystroglycan, and laminin [31, 99], suggesting that AQP4 is implicated in the ability of astrocytes to preserve BBB integrity. In the intracellular region, AQP4 is anchored to several proteins of the astrocyte cytoskeleton, such as α1-syntrophin and dystrophin [3, 26, 62, 93].

The role of AQP4 has been studied using various mouse models, including the dystrophin-null mdx-βgeo transgenic mouse, the α-syntrophin-null [2, 26, 93], and the AQP4 knock-out mouse. Deletion of one of the dystroglycan complex proteins, α-syntrophin, results in failure of AQP4 colocalization in the plasma membrane without alteration of overall AQP4 protein expression [62]. In mdx and syntrophin knock-out mice, astrocyte swelling may be caused by defective water elimination due to impaired AQP4 organization at the plasma membrane [7]. AQP4 knock-out mice provide strong evidence for the involvement of AQP4 in brain water balance in the various types of edema [11, 72, 90, 95, 110]. Although AQP4-deficient mice show no obvious neurological abnormalities, they show considerably reduced brain swelling following the induction of cytotoxic edema after acute ischemic stroke, water intoxication and bacterial infection, indicating that AQP4 has a protective role against the development of brain edema [54, 71]. Primary astrocyte cultures from AQP4 knock-out mice exhibit greatly reduced osmotic water permeability compared with wild-type mice [88]. An additional indication of the function of perivascular AQP4 was obtained by platelet-derived growth factor B (PDGF-B) knock-out mice, which showed abnormal vascular morphogenesis resulting in the absence of pericytes, and presence of endothelial hyperplasia [33].

In biomolecular studies conducted to gain insights into transduction signaling pathways astrocyte end-foot AQP4 inactivation was evaluated *via *protein kinase (PKC) phosphorylation [4] using two potent PKC activators: phorbol 12 13 dybutirate (PD) and phorbol 12 myristate 13 acetate (PMA). The Authors found that intrathecal application of PMA reduced water content and sodium, lending support to the notion that astrocytic end-feet provide the pathway for sodium and obligatory water in the edematous process. In another biomolecular study the 21–nucleotide small interfering RNA duplex (siRNA) was used to suppress AQP4 expression in primary astrocyte cultures. AQP4 knock-out resulted in impaired cell growth, altered cell morphology, and drastically reduced membrane water permeability. Gene expression profiles were analyzed by DNA microarray experiments to identify gene expression patterns perturbed by AQP4 gene silencing. Gene silencing induced upregulation of NGF-B and c-fos, both members of the immediate early gene family encoding transcription factors, also induced by ischemia [49]. Interestingly, three genes (GLUT1, hexokinase, and metallothionein-1) that were downregulated as a consequence of AQP4 gene silencing have been demonstrated to be directly involved in brain ischemia [63].

Moreover, enhanced AQP4 expression is found in reactive astrocytes in cerebral infarction ischemic lesions, suggesting a compensatory upregulation of AQP4 to counter the water imbalance [5, 6].

## 4 AQP7

Data on AQP7 expression in the brain are scanty; the few recent findings regard its expression in perinatal mouse brain development. Shin and co-workers [83] demonstrated AQP7 immunoreactivity in CP throughout brain development and in ependyma, pia and blood vessels during postnatal brain development, surmising its involvement in CSF production.

## 5 AQP9

AQP9 expression has been documented in glial cells, particularly tanycytes and astrocytes [8, 9, 22], endothelial cells of subpial vessels [9], and neurons [9, 18], but its functional role in the nervous system is just beginning to be explored. AQP9 is found in astrocytes, where its expression is upregulated after ischemia [8].

## 6 AQUAPORINS AND STROKE

The presence of AQP4 at the brain-fluid interface suggests that it is important for the brain water balance and may play a critical role in edema [61].

Stroke and vascular brain disease are generally due to cerebral vessel occlusion. The interruption of flow in a brain vessel initiates a chain of events involving cytotoxic edema with cell swelling, followed by BBB leakage and hemorrhagic conversion of tissue [86]. The therapeutic management of edema in stroke is currently limited to hyperosmolar solutions, to reduce brain water content, and decompression craniotomy, to decrease ICP.

Recently, the discovery of AQPs has generated new hypotheses to explain the molecular mechanisms of edema and water transport in the brain [1].

AQP expression (AQP3, AQP5 and AQP8) was detected by Yamamoto and colleagues [106] in neurons and oligodendrocytes, but not in microglia. In an *in vitro* model of hypoxia they demonstrated that expression of AQP4, AQP5 and AQP9, but not of AQP3 and AQP8, was decreased in cultured CNS astrocytes. AQP5 expression was unique in showing a transient up-regulation and subsequent down-regulation after re-oxygenation following hypoxia, whereas both AQP4 and AQP9 recovered albeit to no higher than control levels. The water permeability of AQP4 has been seen to be greater than that of AQP5 [108]. AQP4 knock-out mice display reduced cerebral edema in response to water intoxication and stroke and improved clinical indices of survival and neurological status [53]. These data outline a large role for AQP4 in brain edema; they also suggest that another AQP, one that is hypoxia-responsive (AQP5 or AQP9) and/or non-responsive (AQP3 or AQP8), may affect the rate of progression of edema due to ischemia or other brain injury.

Stroke is divided into hemorrhagic and ischemic. The former is characterized by a hydrostatic effect due to intracerebral hemorrhage, followed by formation of perifocal edema. Edema is sustained by the release of clot-derived proteins and vasoactive substances with direct and indirect actions on BBB integrity [105]. As shown in animal models, injection of blood into the brain induces edema formation through the activation of thrombin, plasminogen activator and urokinase [58], leading to inflammatory cell activation, BBB impairment and eventually scar formation. BBB disruption causes vasogenic edema. After the first 10-20 days the inflammatory mechanisms activated by BBB dysfunction induce CNS cell disruption and red blood cells lysis, with release of hemoglobin and its derivatives. These substances compound the cell damage, with secondary cellular injury that gives rise to cytotoxic edema [28]. Subsequently, the production of interleukin, metalloproteinases and other degradation products maintains and contributes to both cytotoxic and vasogenic edema, which last 2-3 weeks [79]. Hyperosmolar solutions are the only therapeutic option for edema in intracerebral hemorrhage, even though their effects in preventing mortality and morbidity are not universally acknowledged. The scope for using AQPs as possible therapeutic targets to reduce edema in cerebral hemorrhage thus deserves extensive investigation.

Ischemic stroke is usually due to the occlusion of a cerebral artery by a thrombus. This event induces various cell modifications, including cell swelling due to cytotoxic edema, BBB disruption with vasogenic edema, and finally conversion of ischemic into hemorrhagic tissue. The BBB dysfunction is related to impaired Na/K ATPase function resulting in reduced ATP synthesis and intracellular sodium accumulation. Lactate levels rise in response to cellular ischemia. Together, lactate and sodium induce a water gradient toward the intracellular space, with formation of cytotoxic edema. The BBB disruption has been hypothesized to be related to mechanisms such as reverse pinocytosis [15] and calcium modulation signaling [14]. The role of AQPs has also been explored in animal models of ischemic stroke to gain insights into the pathophysiology of cerebral edema.

Yan and colleagues [107], studying the expression of Na^+^, K^+^, Cl^- ^cotransporter, suggested that AQP4 expression is initially induced by changes in the metabolic environment, and that its upregulation is related to compensation of the osmolarity gradient by water transport into the brain. Continued AQP4 expression even after BBB disruption may exacerbate subsequent brain edema, as suggested by another study demonstrating that development of brain edema was greatly attenuated in AQP4 knock-out mice [54].

The expression profiles of AQP4 and AQP9 in edema were recently studied at various time points after mouse transient cerebral ischemia to gain insights into their role. Two peaks of AQP4 expression were observed 1h and 48h post stroke, coinciding with the two peaks of hemispheric swelling [18]. This time course was at variance with brain trauma data, where AQP4 levels decreased in the first 48h then rise again [43-45]. The discrepancy suggests that AQP4 has a complex role in edema formation and resolution. Unlike AQP4, AQP9 showed a gradual and significant induction at 24h, with no correlation to swelling, suggesting that AQP4, but not AQP9, plays a role in edema formation after transient cerebral ischemia in mice. Interestingly, AQP4 expression was rapidly regulated with a major induction 1h after stroke in astrocyte end-feet, indicating that early time points after the onset of the brain disorder should be considered [18].

A recent investigation of AQP4 modulation after 3h middle cerebral artery (MCA) occlusion and 30 min reperfusion in rat demonstrated peak AQP4 expression at 72h, suggesting that delayed upregulation could play a significant role in brain water clearance after ischemic edema. Friedman and co-workers applied 1 to 8h of MCA occlusion followed by 30 min reperfusion and documented a rapid, spatially selective loss of AQP4 immunoreactivity in regions subjected to sufficiently severe ischemic injury [25].

The experimental data on AQP4 regulation from mouse stroke models are contradictory; some studies reporting significant upregulation after 30 min MCA occlusion and 1-48 h reperfusion [78] and others describing a decrease in AQP4 immunoreactivity following longer MCA occlusion, indicating that its loss post stroke may require a critical duration or severity of ischemic injury.

## 7 AQPS AND THE CHOROID PLEXUS

CSF fluid provides physical support for the brain and a specialized extracellular environment facilitating the transport of nutrients, peptides, and hormones to the brain [41]. CSF is held to form predominantly in the cerebral ventricles; other potential sites include CP, brain parenchyma, and the ependymal lining of the ventricles [16, 59]. CSF produced by CP epithelial cells (the majority) and by extrachoroidal tissues flows through the ventricles to sites of drainage in the subarachnoid space [17].

The CSF secretion mechanisms are well described in the adult, whereas much less is known about early CP development. Secretion may start with CP formation. In the rat the lateral ventricular CP arises around embryonic day (E) 14 [21, 41]; AQP1 is found in CP epithelial cells from E15 with an adult pattern [41], strongly suggesting the beginning of CSF formation at this time.

Finely regulated CSF composition is vital for the brain [38, 39, 77]. Aging- or disease-induced changes in its circulation adversely affect neuron performance [77, 84]. Multiple conditions, e.g. tumours, infections, trauma, ischemia and hydrocephalus, may influence CP-CSF dynamics [24, 40, 48, 73, 101].

Hydrocephalus is the result of an imbalance between CSF production and resorption that induces an expansion of the ventricular system and increased ICP [91]. It is usually caused by obstruction to CSF flow in the ventricular system or subarachnoid space [55] The increased ICP drives flow from the ventricles to the parenchyma, leading to extracellular edema, especially in subventricular white matter [103].

AQP channels, which facilitate water diffusion across the blood-CSF interface [12, 89], appear in developing CP epithelia [29, 41] and are retained throughout life. AQP1 is heavily expressed at the ventricular-facing membrane of CP epithelia [47, 104] but not in BBB endothelium. Water movement from blood to cerebral ventricles may thus be selectively modulated by regulatory phosphorylation or ubiquitination of AQP1 [32, 46].

AQP1 null mice show a 25% reduction in the rate of CSF secretion, reduced osmotic permeability of CP epithelia, and decreased ICP [69].

A recent study has also documented that AQP4 is upregulated in periventricular white matter of hydrocephalic rats, and that upregulation increases with disease severity, supporting the existence of an adaptive response aimed at clearing excess fluid [91].

Obstructive hydrocephalus, produced by kaolin injection into the cisterna magna, induced faster ventricular enlargement in AQP4 knock-out than in wild-type mice [11]. The diminished water permeability of ependymal layer, subependymal astrocytes, astrocytic foot processes and glia limitans produced by AQP4 deletion reduces CSF elimination through these routes.

All these findings support a role for AQP1 in facilitating CP secretion of CSF into the cerebral ventricles, and support the hypothesis of a role for AQP1 inhibitors in treating hydrocephalus and benign intracranial hypertension, which are associated with CFS formation or accumulation [61]. Theoretically, the same goal could be achieved by enhancing the elimination rate of CSF by up-regulating astrocytic expression of AQP4 [60].

Significantly for CSF dynamics, AQP1 expression is diminished in late life. Thus, 20-month-old Sprague-Dawley rats display substantially reduced AQP1 expression in CPE than their young adult counterparts [57]. Accordingly, CSF formation is less abundant in senescent rats [75]. Human CP epithelium expresses AQP1 [52]. This raises the possibility of treatments that upregulate or restore AQP1 expression in aged humans, whose CFS turnover is damaged by Alzheimer’s disease or normal pressure hydrocephalus [84, 85], by applying an opposite therapy to the one used to manage hydrocephalus.

MRI techniques affording better resolution now allow diffuse villous hyperplasia of the CP to be clinically recognized as a potential, though rare, source of non-obstructive hydrocephalus [13, 27, 36].

## 8 AQP1 AND TUMOURS

An additional role of AQPs connected to their water-transporting function is related to cell migration [97]. AQP1 is expressed in tumour microvessels [23], where it seems to contribute to increased BBB water permeability in aggressive brain tumours [70].

The involvement of AQPs in cell migration was uncovered based on the observation that tumour angiogenesis was impaired in AQP1-null mice and upon subsequent characterization of endothelial cell cultures from wild-type and AQP1-null mice [82]. Verkman and co-workers [97] proposed a mechanism of AQP-facilitated cell migration whereby actin cleavage and ion uptake at the tip of the lamellipodium create local osmotic gradients that drive water influx, facilitating lamellipodial extension and cell migration. AQP-facilitated cell migration was also detected in tumour cells [37], suggesting that it may have an important role in tumour angiogenesis and spread, and accounting for AQP expression in many tumour cell types and for correlations found between AQP expression and tumour stage in some tumours [97].

AQP regulation in human CNS tumours has extensively been studied, usually on limited numbers of samples. Endo and colleagues [23] demonstrated AQP1 immunoreactivity in glioma cell lines implanted in rodents. Differential gene expression analysis identified AQP1 upregulation in four cases of primary human glioblastoma multiforme [56]. Saadoun and co-workers [80] confirmed AQP1 up-regulation in high-grade astrocytomas using immunohistochemical techniques. AQP4 also seems to be up-regulated in glioblastoma [81]. AQP4 over-expression in human astrocytoma correlates with detection of brain edema on MRI [81, 100].

Oshio and colleagues [68] documented intense AQP1 upregulation in 36 human glial tumours using RT-PCR, Western blotting and immunohistochemistry and demonstrated that AQP1 localization was primarily or exclusively to the membrane of neoplastic astrocytes. The exact mechanism of its up-regulation in cells that do not normally express it is unknown; possible mechanisms include dedifferentiation and loss of cell type-specific transcriptional regulation. The involvement of AQPs in transmembrane water transport and their up-regulation in neoplastic glial membranes suggest a potential role for them in the formation of tumour associated edema [68].

CP papilloma and carcinoma are rare brain neoplasms often associated with obstruction of CSF ventricular pathways and rarely with communicating hydrocephalus due to CFS hyperproduction by CP tumour [10, 19]. These tumours show different patterns of AQP1 expression [50]. Villous hypertrophy of CP is also associated with overproductive hydrocephalus. In particular, AQP1 expression is strongly though not uniformly increased in some benign papilloma lesions with a preserved morphological structure, predicting CSF hypersecretion, while complete loss of the papillary architecture in CP carcinoma entails a lack of AQP1 expression, which is associated with normal ventricular size or with obstructive hydrocephalus. Thus, preservation of tight junctions and persistent expression of proteins associated with specialized features, e.g. AQP1, readily differentiate papilloma from carcinoma, possibly explaining why the latter is not associated with hypersecreting hydrocephalus [50].

AQP1 expression was analyzed in 10 surgical specimens of hemangioma of the CNS, which becomes clinically apparent through the development of huge cysts. Stromal cancer cells displayed high AQP1 expression, while large cyst volume correlated with higher immunostaining scores [51].

## CONCLUSION

AQPs are a rapidly growing and promising field of research. A broad spectrum of specialized functions is expected to be found for these channels in the CNS. The discovery of selective pharmacological agents for AQPs is a stimulating challenge, especially given the lack of treatments aimed at this family of channels, and the direct and fundamental roles subserved by water channels such as AQP1 and AQP4 in brain fluid homeostasis. AQP4 inhibitors seem to have a neuroprotective effect in cytotoxic edema, while AQP4 activators or upregulators may help clear vasogenic and hydrocephalic edema. AQP inhibitors in tumour cells and microvessels are predicted to reduce angiogenesis and thus tumour spread, offering adjunctive chemotherapy.
